# Perspectives on Robustness and Resilience of Complex Networks

**DOI:** 10.3390/e28030252

**Published:** 2026-02-24

**Authors:** Daniele Proverbio, Stefano Boccaletti

**Affiliations:** 1 Department of Industrial Engineering, University of Trento, 38123 Trento, Italy; 2 CNR, Institute of Complex Systems, Via Madonna del Piano 10, 50019 Sesto Fiorentino, Italy; stefano.boccaletti@isc.cnr.it

## 1. Introduction

Traditionally, the analysis of physical and dynamical systems has focused on the question of how would a system, starting from an initial condition, evolve in time, and whether it would settle on a specific pattern, or regime [1]. Motivated by observations in the natural and engineering sciences [2], a complementary question emerged, i.e., under which conditions systems subject to uncertainties, perturbations or fluctuations manage to preserve their operating regime(s) or key properties over time.

This second question is addressed by the concepts of robustness and resilience, which concern the capability of a complex system to preserve its regimes and properties despite various types of uncertainties, fluctuations and perturbations. Robust and resilient behaviours can be recognised in multiple systems in nature, society and engineering, from protein transcription [3] to cell swarming [4], from circadian rhythms [5] to neural signalling [6], as well as in ecological [7], social [8], financial [9] and technological systems [10]. In all these cases, the system of interest is able to carry on its function despite parametric uncertainties, dynamical disturbances such as stochastic noise, or topological alterations in their network structure. Also, an additional question has taken the spotlight in theoretical and applied fields: in case a system does *not* manage to withstand uncertainties and perturbations, and is therefore likely to undergo a regime shift [11], is it possible to extract early-warning signals to alert in advance about losses of robustness or resilience [12,13]?

Recently, renovated interest has emerged in the scientific community around these topics, with particular attention on the robustness and resilience of complex networks and dynamical systems. This interest is demonstrated by a set of influencing surveys recently published, covering complementary perspectives. Liu et al. [14] focused on network resilience as a consequence of bifurcations brought about by changing parameters of dynamical networks; Artime et al. [15] provided a heuristric take on robustness and resilience focusing on the network structure and topology, including targeted attacks as sources of disturbances; Krakovskà et al. [16] surveyed popular metrics to measure and quantify resilience and loss thereof in dynamical systems; and Proverbio et al. [17] surveyed formal definitions of robustness and resilience for dynamical networks, which also include the intuitive definitions considered in [14,16] as particular cases, with additional applications of related early warning signals.

Despite the growing interest, both theoretical and applied, many research gaps remain, impeding the building of a coherent and cohesive research framework. As already noted in [18,19], a common terminology is currently missing when referring to the meaning and operational measures of robustness and resilience, with fields such as network theory, theoretical ecology, physics, and engineering using different nuances of their associated intuitive definitions, while mathematically based formal definitions, outlined, e.g., in [14,17,20], are still being uptaken, slowing down interdisciplinary collaborations. The interplay of analytical and computational investigations is also under development, as they require the further development of formal methods and the integration of nonlinear dynamics [21] and complex network theory [22]. Applications of theoretical methods to real-world scenarios is also an area of active development, as particularly demonstrated by the contributions of this Special Issue (cf.Section 2). Finally, there is a question as of how to extend robustness and resilience definitions to possibly any property of interest for a dynamical network, beyond stability and regime preservation.

Fuelled by compelling research questions and the need to bring together diverse perspectives on the topic, the present Special Issue called for contributions, both theoretical and applied, that can help frame a comprehensive picture of the quantitative theories and techniques developed and deployed to better understand and predict the key mechanisms of dynamical networks persisting in their functions despite alterations.

## 2. Contributions

This Special Issue received five contributions, well representative of the diverse focus associated with the topics of robustness and resilience: developing methods to *assess* them, or *using* these concepts to enhance some properties of interest; and focusing more on the development of formal or heuristic methods and metrics, or applying them on real-world problems.

Contribution 1 deals with optimizing the robustness of an energy system (a grid of wind power plants), quantified in terms of reduced loss in case of wind uncertainty and targeted attacks on communication nodes among plants. Here, the authors take an engineering perspective on robustness, in terms of capability to withstand parametric uncertainties (in this case, upon the input) and node failures so as to maintain the power production capabilities. The article is a typical example of robustness analysis in practice, conducted on a multi-layer network and based on the quantitative definition of the quantities preserve, coupled with simulation and optimization studies.

Contribution 2 provides an interesting application of information-theoretic measures, computed after reconstructing the stock market high-order network, that can be informative about the potential significant events emerging from stock dynamics. The article represents a key example of a model–data-coupled study for the monitoring of regimes: first, extract the most likely structure underlying a system of interest (in this case, the higher-order network that best represents stock markets beyond pairwise interactions); then, compute advanced statistics to capture noteworthy dynamics. Future studies may strengthen the association of information-theoretic metrics and formal definitions of resilience, to better support the use of such metrics as detectors of resilience loss for financial dynamics.

Contribution 3 focuses on maintaining normal operations of critical infrastructures against targeted attacks. In this sense, the article primarily takes a network-based definition of robustness, meant as the capacity to carry on normal functions against perturbations on the topology of the infrastructure. Dynamics is then introduced using a game-theoretic scenario between attackers and defenders. Robustness and cascading failures are then tracked using performance metrics, subsequently tracked during simulation experiments, both synthetic and based on real-world network counterparts.

Contribution 4, instead of promotingthe resilience of a network, uses it as an interpretativetool to better understand the complex web of mutualistic interactions in an ecological system, identifying possibly “core” and “redundant” species for the survival of the ecological network. After defining a dynamical network model to represent the interactions between species with different roles, the author use the bifurcation-based definition of resilience, similarly to Liu et al. [14], to identify, based on computational studies, which species are more “essential” or “redundant” for the preservation of the bifurcation structure or the encounter of a tipping point towards ecosystem collapse. This contribution outlines how properties such as robustness and resilience can be used to gain insights onto the key survival or evolutive mechanisms of complex systems.

Contribution 5, similarly to Contribution 3, addresses the problem of preserving core properties of a network against attacks or unexpected events. In this case, the authors identify a set of performance metrics, of interest for the function of hyper-networks, and devise an optimization method to improve the function maintenance. Like others, this study relies on measurable quantities, such as centrality-based metrics, that are associated with desired functions of a hyper-network, and use them as proxies of network robustness (even in this case, primarily in a network sense, i.e., the ability to withstand topological alterations). Based on simulation experiments, both on synthetic and empirical networks, the authors carry out an optimization procedure aimed at better controlling complex networks through their robustness-related metrics.

## 3. Conclusions and Future Directions

In addition to the specific content of each SI paper, the demography of the contributions is also informative of the research community. Interestingly, this Special Issue has mostly intercepted authors from network science or control engineering, who then applied their expertise to “on-field” problems such as ecology, finance or power grid management. This observation signals the necessity to foster spaces for interdisciplinary dialogue so as to bring forth a mutual collaboration between formal and applied studies.

Overall, this Special Issue has shown that multidisciplinary endeavours should be dedicated to unraveling the key characteristics—structural, mechanical, or dynamical—that guarantee robustness and resilience, to develop comprehensive frameworks to study them, and to detect and anticipate losses of robustness and resilience. Cross-fertilization among communities (from complex systems physics, engineering, control, mathematics, or applied sciences) are warranted to bring together expertise and build a coherent research field based on shared common definitions and methodologies. In addition to the current higher focus on the analysis and understanding of robustness and resilience (cf.Contributions 1, 3), additional research avenues in the direction of real-time monitoring of fragile systems (aligned with Contribution 2), use of resilience to gain information about systems’ adaptation (Contribution 4) or management and control [23] (see also Contribution 5) bear potential to significantly advance the significance of robustness, resilience and early-warning studies for real-world applications.

Together with recent surveys, articles and dedicated workshops at top conferences, this Special Issue brings a strong signal about the growing importance of studying how systems thrive in the face of perturbations and uncertainties, fostering the development of advanced theoretical frameworks and applied methodologies to better understand, interpret, predict and control complex systems and networks in nature, society and technology.

## Data Availability

No new data were created or analyzed in this study. Data sharing is not applicable to this article.
